# Rational Design of Peptides Derived from Odorant-Binding Proteins for SARS-CoV-2-Related Volatile Organic Compounds Recognition

**DOI:** 10.3390/molecules27123917

**Published:** 2022-06-18

**Authors:** Jin Wang, Kenji Sakai, Toshihiko Kiwa

**Affiliations:** Faculty of Interdisciplinary Science and Engineering in Health Systems, Okayama University, 3-1-1, Tsushima-Naka, Kitaku, Okayama 700-8530, Japan; sakai-k@okayama-u.ac.jp (K.S.); kiwa@okayama-u.ac.jp (T.K.)

**Keywords:** rational design, odorant-binding protein, peptide, SARS-CoV-2, volatile organic compounds, computational tools

## Abstract

Peptides are promising molecular-binding elements and have attracted great interest in novel biosensor development. In this study, a series of peptides derived from odorant-binding proteins (OBPs) were rationally designed for recognition of SARS-CoV-2-related volatile organic compounds (VOCs). Ethanol, nonanal, benzaldehyde, acetic acid, and acetone were selected as representative VOCs in the exhaled breath during the COVID-19 infection. Computational docking and prediction tools were utilized for OBPs peptide characterization and analysis. Multiple parameters, including the docking model, binding affinity, sequence specification, and structural folding, were investigated. The results demonstrated a rational, rapid, and efficient approach for designing breath-borne VOC-recognition peptides, which could further improve the biosensor performance for pioneering COVID-19 screening and many other applications.

## 1. Introduction

As of 2022, the COVID-19 pandemic has lasted for more than two years, resulting in enormous worldwide damages and crises. The world population has suffered from this dangerous and unpredictable viral disease due to insufficient preparation, a combination of a lack of rapid screening and detection, and low availability of therapeutic drugs and highly efficient vaccines. The new disease has challenged our current knowledge and techniques and forced us to acquire vital information faster and more accurately. Scientists and engineers have focused on developing portable polymerase chain reaction (PCR) devices, test kits, vaccines, and virus-inactivation instruments to end the pandemic and return life to normal as soon as possible [1,2,3,4]. Since the pandemic, numerous transducers and receptors have been identified for SARS-CoV-2 detection [5,6,7,8,9]. SARS-CoV-2 virus, nucleic acids and antigens are usually recognized as diagnostic indicators. Recently, the SARS-CoV-2-related breath samples were explored as novel biomarkers. The U.S. Food and Drug Administration (FDA) has authorized the very first emergency approval for diagnostic COVID-19 assays using breath-borne volatile organic compounds (VOCs) [10].

Insect odorant-binding proteins (OBPs) have an outstanding ability to recognize various kinds of VOCs [11,12,13,14,15]. Although expressed at high levels in insects, these OBPs have not been fully characterized and developed as biosensors owing to difficulties in their structural identification and high-cost synthesis. Short-chain peptides have been designed as promising molecular recognition elements and utilized in biosensors for detecting a range of targets, including proteins, viruses, bacteria, and small molecules [16,17,18,19]. Phage-display library technology is a widely approach used for peptide synthesis and selection for ligand–molecule binding with random amino acid sequences [20,21]. Many studies have focused on the rational or computational design of specific peptides for small ligand molecule recognition [22,23,24,25,26]. In our previous investigation, a small-molecule-binding peptide derived from a complementary determining region (CDR) in a monoclonal antibody was rationally designed to detect nitroaromatic compounds with high sensitivity and selectivity [19,27]. Furthermore, when coupled with single-walled carbon nanotubes (SWCNT), the peptide-SWCNT hybrid material offers more improved properties [20,28]. 

Therefore, in this study, robust computational docking and prediction tools were used to identify and characterize OBPs and peptides. We aimed to apply computational tools to identify the structural properties of OBPs-ligand and rationally design the SARS-CoV-2-related VOC-recognition peptides derived from insect OPBs for pioneering biosensor development.

## 2. Results

**Figure 1:**
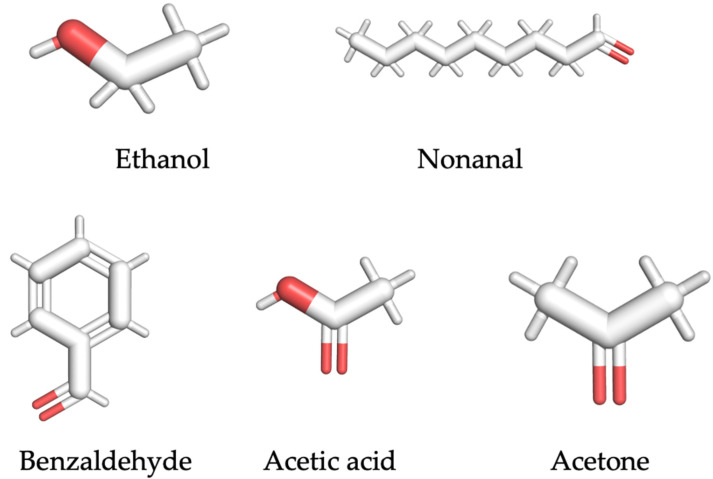
Structure of SARS-CoV-2-related VOC biomarker.

Based on recent literature, five representative SARS-CoV-2-related VOC biomarkers, ethanol, nonanal, benzaldehyde, acetic acid, and acetone from exhaled breath samples, were selected [17,29,30,31,32]. The structures of these VOC biomarkers are shown in Figure 1. In addition, the information and properties of each VOC ligand, including the molecular weight, odor description, and vapor pressure, are summarized in Table 1. 

**Figure 2:**
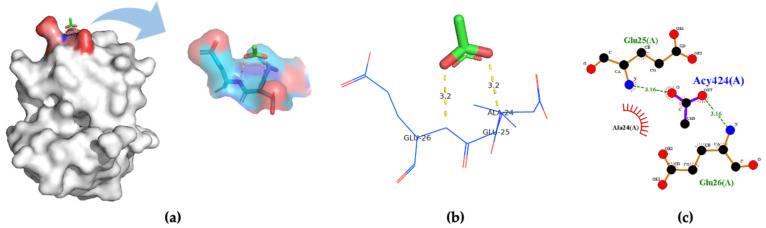
(**a**) 3D visualization of the docking model; (**b**) binding residues identification; (**c**) 2D ligand–-protein interaction diagrams of AgamOBP20 (PDB ID:3VB1) bound to acetic acid.

**Figure 3:**
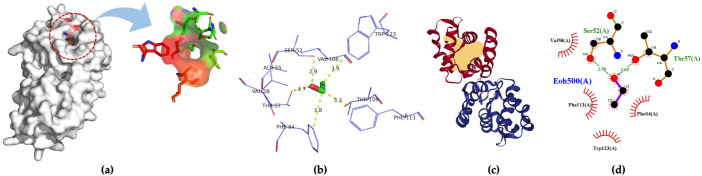
(**a**) 3D visualization of the docking model; (**b**) binding residues identification; (**c**) best position of predicted binding pocket; (**d**) 2D ligand–protein interaction diagrams of odorant binding protein LUSH from *Drosophila melanogaster* (PDB ID:1OOF) with ethanol.

**Figure 4:**
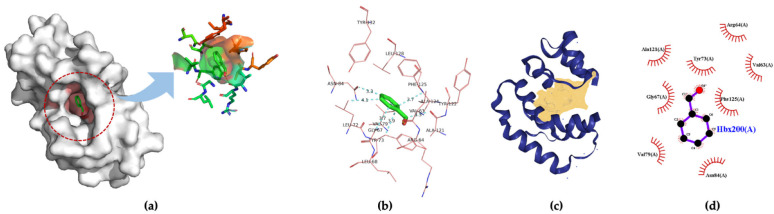
(**a**) 3D visualization of the docking model; (**b**) binding residues identification; (**c**) best position of predicted binding pocket; (**d**) 2D ligand–protein interaction diagrams of AgamOBP22a (PDB ID:3L4L) with benzaldehyde.

Three-dimensional structures of four insect OBPs were obtained from RCSB Protein Data Bank (PDB). One insect OBP was obtained from the mosquitoes OBP database with identified amino acids sequence information. *Anopheles gambiae* AgamOBP20 (PDB ID:3VB1) was used for acetic acid binding, AgamOBP22a (PDB ID:3L4L) for benzaldehyde recognition; and AgamOBP47 (PDB ID:3PM2) for acetone binding. *Aedes aegypti* Aaegobp39 were used for nonanal recognition, and the OBP LUSH from *Drosophila melanogaster* (PDB ID:1OOF) was selected for specific ethanol binding, respectively. Figure 2 shows the docking and visualization results for OBP AgamOBP20 in the open stage with the acetic acid–ligand complex. We found that the GLU25 and GLU26 amino acids played a very important role in forming the hydrogen bonds (with a distance of 3.2 Å). In contrast, ALA24 formed hydrophobic interactions with acetic acid based on the binding residues identification and interaction analysis (Figure 2a–c). The ALA-GLU-GLU amino acids sequence was identified as a binding site for the recognition of acetic acid. LUSH, a non-enzyme protein, specifically bonds to alcohol (Figure 3a). Strong hydrogen bonds (polar contacts) were formed between ethanol and SER52 and THR 57 amino acids (with distances of 2.9 Å and 2.7 Å) (Figure 3b,c). However, THR48 was not included in the AutoDock model after the prediction results were compared with the experimental analysis [33]. A group of amino acids (polar and non-polar contacts) comprised a hydrophobic pocket in the chain A of LUSH (Figure 3a,c; Table S1). The 2D ligand–protein interaction diagrams displayed a network of ethanol-binding sites (Figure 3d). As such, a nine-amino-acid oligopeptide sequence (SER-ALA-THR-VAL-PHE-VAL-THR-PHE-TRP) that specifically recognizes ethanol was identified in the LUSH protein. The benzaldehyde-OBP complex model was investigated, and the binding residues were revealed (Figure 4 and Table S2) using the same approach. Hydrogen bonds formation by VAL63 (3.0 Å),and the π stacking between PHE125 and benzaldehyde were also observed (with a distance of 5.3 Å, angle 74.7°). This feature relates to the fact that the aromatic amino acids TRP, PHE, or TYR always play a key role in interactions with aromatic ligands. 

**Figure 5:**
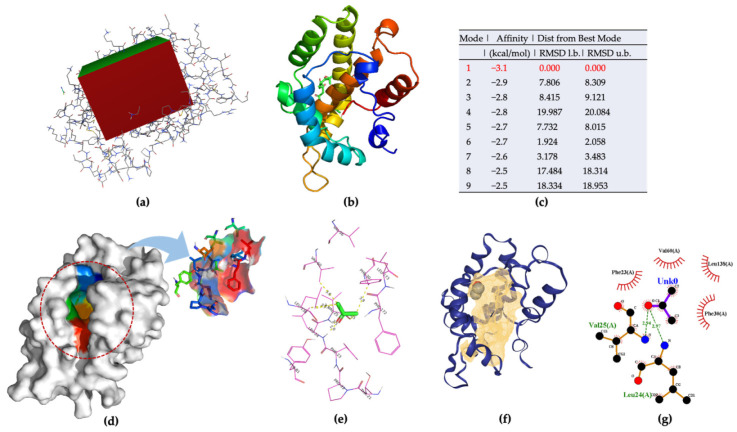
(**a**) Grid box for AgamOBP47–acetone docking model; (**b**) nine docking models predicated using AutoDock Vina; (**c**) ranking results of docking model; (**d**) 3D visualization of the best docking model; (**e**) Binding residues identification; (**f**) best position of predicted binding pocket; (**g**) 2D ligand–protein interaction diagrams of AgamOBP47 (PDB ID:3PM2) with acetone.

AgamOBP47 belongs to the C-plus class of OBPs. As it is a novel type of OBPs, with a longer sequence than classical OBPs, it provides a shallow channel with sufficient space to accommodate the odorant ligands. The ligand-binding site of AgamOBP47 located between the core and the additional domains were demonstrated in the experimental results. The X-ray structure of AgamOBP47 was used for acetone–ligand docking. All docking and all required parameters were performed using Autodock Vina. A docking grid box, measuring 52 × 42 × 58 in the x-, y-, and z- directions, was set (Figure 5a). The nine docking poses, and mode ranking scores are shown in Figure 5b,c and Figure S2, respectively. Docking mode 1, with a more favorable affinity (−3.1 kcal/mol) than the other models, was chosen as the best docking mode for further analysis based on the docking calculations. The binding pocket and amino acid residues were visualized (Figure 5d–f and Table S3). Twelve amino acids around the acetone ligand forming hydrogen bonds (LEU24 and VAL25) and hydrophobic interactions were selected as polypeptide sequences for acetone binding.

**Figure 6:**
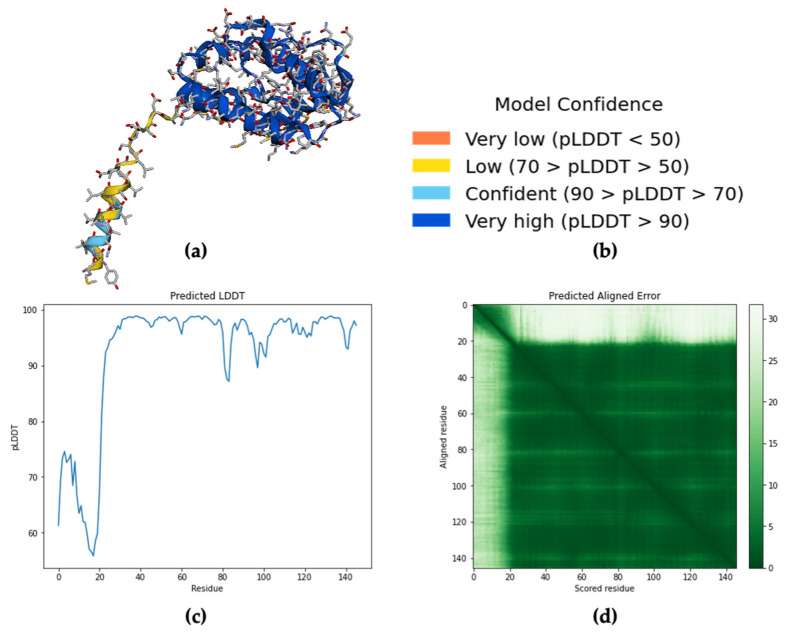
(**a**) Prediction results of nonanal-binding protein *Aedes aegypti* Aaegobp39 using AlphaFold Colab; (**b**) Model confidence; (**c**) The general the predicted local-distance difference test (pLDDT) for intra-domain confidence; (**d**) Predicted aligned error (PAE) for determining between domain or between chain confidence.

**Figure 7:**
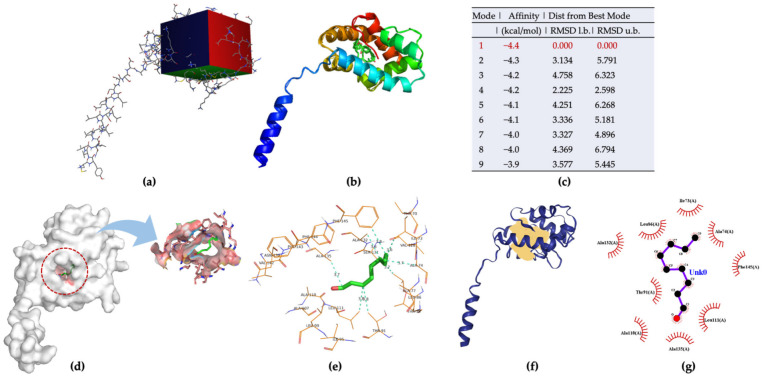
(**a**) Grid box for Aaegobp39-nonanal docking model; (**b**) nine docking modes predicted using AutoDock Vina; (**c**) ranking results of docking model; (**d**) 3D visualization of the best docking model; (**e**) binding residues identification; (**f**) best position of predicted binding pocket; (**g**) 2D ligand–protein interaction diagrams of Aaegobp39 with nonanal.

*Aedes aegypti* Aaegobp39 is a strong aldehyde-binding mosquito OBPs (Figure S1). However, the crystal structure of Aaegobp39 was not identified. The machine learning-based approach AlphaFold has been reported as a highly precise prediction tool for protein structure compared with the conventional methods including X-ray and cryogenic electron microscopy (cryo-EM) [34,35]. Based on the sequence information (Figure S1), the prediction model of the nonanal-binding protein Aaegobp39 is displayed in Figure 6. The prediction shows high model confidence in the predicted local-distance difference test (pLDDT) and low residue-predicted-aligned error (PAE). This indicates high accuracy in relation to the OBP structure, making it suitable for nonanal docking. The docking parameter files were set, and the grid box dimensions were 52 × 42 × 58 in the x-, y-, and z-directions (Figure 7a). Nine docking positions were obtained (Figure 7b and Figure S3). The docking model ranking is shown in Figure 6c. Mode 1, with an affinity for nonanal of −4.4 kcal/mol, was considered the best mode for the ligand–Aaegobp39 complex. Analyzing the 3D visualization docking results and predictions in Figure 7d–g and Table S4, no hydrogen bonds were formed, and the hydrophobic interaction played an important role in nonanal binding. The 21-amino acid peptide was selected as the final nonanal-binding residue based on pocket characterization and distance measurement.

Table 2 summarizes the properties of the VOC–ligand binding peptides. Structures exhibiting grand average of hydropathicity (GRAVY) scores above 0 were considered hydrophobic peptides.

**Figure 8:**
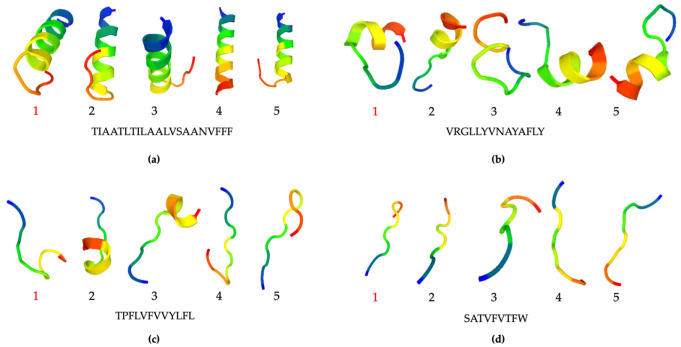
Best five models (representatives of five best clusters) of peptide structure prediction using PEP-FOLD according to the clustering reports: nonanal- grand-average-of-hydropathicity-binding peptide (**a**); benzaldehyde-binding peptide (**b**); acetone-binding peptide (**c**); ethanol-binding peptide (**d**).

An instability index value of less than 40 was considered to indicate a stable structure. Thus, the calculation results indicated that the binding peptides for nonanal, benzaldehyde, acetone, and ethanol were stable, whereas the acetic acid sequence was too short for prediction. The five best models (representatives of the five best clusters) for peptide structure prediction using PEP-FOLD were generated in the alpha helix, coil, or extended structural conformation (Figure 8 and Figure S4) [36,37,38]. According to the clustering score reports (Tables S5–S8), Model 1 was selected as the best for the predicted peptide structures. Furthermore, the helical nonanal-binding peptide structure was predicted by AlphaFold, similar to model 4 generated by PEP-FOLD (Figure S5). The results showed that different peptide sequences revealed different conformational states that may contribute to VOC-binding sensitivity and specific selectivity.

## 3. Discussion

Peptides mimicking OBPs are considered promising method and are widely used in biosensing research. In contrast to phage display for peptide screening, rational design of VOC-binding peptides derived from insect OBPs could be an efficient approach for sensing-element synthesis. Although the core binding residues have been included in the sequence of each peptide, the length of the peptide sequence may need further analysis by molecular dynamics simulation and calculations for optimization and the experimental results. The use of powerful computational tools and machine learning approaches could further pave the way for specific peptide design and for SARS-CoV-2-related VOC sensor device development. Several previous investigations have demonstrated the validity of this approach in experimental analysis [39,40]. Furthermore, cyclic peptides or peptide matrixes having a novel conformation may be designed by adding a cysteine amino acid to form disulfide bonds and thereby improve the affinity of the target VOCs. Specifically, three phenylalanine (F) amino acids in the nonanal-binding sequence may form π–π interactions with the SWCNT that could improve recognition performance. Moreover, a peptide-array-based analysis platform coupled with machine-learning algorithms may enhance discrimination ability. 

## 4. Materials and Methods

The properties of five VOC biomarkers: ethanol, nonanal, benzaldehyde, acetic acid, and acetone were obtained from PubChem (https://pubchem.ncbi.nlm.nih.gov, accessed on 3 December 2021). Four insect OBPs with 3D crystal structures (three-dimensional structure), AgamOBP20 (PDB ID:3VB1), AgamOBP22a (PDB ID:3L4L), AgamOBP47 (PDB ID:3PM2), and LUSH (PDB ID:1OOF) were obtained from the RCSB Protein Data Bank (PDB). *Aedes aegypti* Aaegobp39 was obtained from the OBP database of mosquitoes with amino acid sequence information. The protein structure of Aaegobp39 was predicted using AlphaFold Colab (DeepMind, Alphabet Inc., Mountain View, CA, USA). AutoDock Vina (version 1.1.2), an open-source program provided by the Molecular Graphics Lab at The Scripps Research Institute (San Diego, CA, USA), was used for ligand–protein complex model docking. PyMOL 2.5 (professional version, Schrödinger, LLC, New York, NY, USA) was used to visualize the docking results and identify binding residues. The binding-pocket position was predicted using the online server Proteins*Plus* (DoGSiteScorer), provided by the ZBH Center for Bioinformatics (https://proteins.plus, accessed on 3 December 2021). 2D ligand–protein interaction diagrams were generated using the LigPlot+ software (European Molecular Biology Laboratory, European Bioinformatics Institute (EMBL-EBI), Cambridgeshire, UK). The peptide conformation was predicted using the PEP-FOLD3 and RPBS Web Portal platform, which is developed by the Institut Pasteur Biology IT Center and the Ressource Parisienne en Bioinformatique Structurale. The peptide properties, including the theoretical pI, grand average of hydropathicity (GRAVY), and instability index, were calculated using the SWISS-MODEL ProtParam tool. 

## 5. Conclusions

Insect OBPs are well-known for their outstanding odorant recognition abilities. However, the use of OBPs as sensing elements remains a challenge. In this study, the rational design of peptides derived from insect OBPs for COVID-19 breath-borne VOC recognition was performed. The short-chain fragments derived from the binding pocket of the insect OPBs were investigated, and the ligand–OBPs interaction mechanism was clarified by computational visualization. As expected, non-covalent interactions, including hydrogen bonds, π stacking, and hydrophobic interactions were observed between the VOC ligands and insect OBPs. Furthermore, the peptide sequences for five representative VOCs were determined, and their conformations were predicted. The approach addressed here may drive or accelerate the development process of a biosensor developed for pioneering SARS-CoV-2 diagnosis. 

## Figures and Tables

**Table 1:** Summary of representative SARS-CoV-2-related VOC ligand.

VOC Ligand	Molecular Formula	Molecular Weight (g/mol)	Odor Description	Vapor Pressure (mmHg)	Flash Point (°F)
ethanol	C2H6O	46.07	Weak, ethereal, vinous odor	59.27	57.2
nonanal	C9H18O	142.24	Orange-rose odor	0.37	147
benzaldehyde	C7H6O	106.12	Odor resembling oil of bitter almond	1.27	145
acetic acid	C2H4O2	60.05	Sour, vinegar-like odor	15.73	103
acetone	C3H6O	58.08	Fruity odor	231.53	1.42

**Table 2:** The properties of the peptide derived from insect OBPs.

VOC Ligand	Peptide Sequence	Molecular Weight (g/mol)	Theoretical pI	GRAVY	Instability Index
nonanal	TIAATLTILAALVSAANVFFF	2154.58	5.19	1.981	18.70
benzaldehyde	VRGLLYVNAYAFLY	1661.96	8.47	0.993	20.14
acetone	TPFLVFVVYLFL	1457.82	5.18	2.400	18.93
ethanol	SATVFVTFW	1057.21	5.24	1.411	13.17
acetic acid	AEE	347.32	-	-	-

The theoretical pI, grand average of hydropathicity (GRAVY) and instability index were canulated by SWISS-MODEL ProtParam tool (https://swissmodel.expasy.org/, accessed on 3 December 2021).

## Data Availability

Raw data that support the findings of this study are available from the corresponding author, upon reasonable request.

## Supplementary Materials

The following supporting information can be downloaded at: https://www.mdpi.com/article/10.3390/molecules27123917/s1, Figure S1: The free binding energy of mosquito classic and Plus-C OBPs corresponding to the nonanal ligand [41]. Figure S2: Nine docking models of AgamOBP47 and ligand acetone complex. Figure S3: Nine docking models of Aaegobp39 and the nonanal ligand complex. Figure S4: Local structure prediction profile for TIAATLTILAALVSAANVFFF (a), VRGLLYVNAYAFLY (b), TPFLVFVVYLFL (c), and SATVFVTFW (d). The profile is presented using the following color code: red: helical, green: extended, blue: coil. Figure S5: (a) Prediction results of nonanal-binding peptide by AlphaFold Colab; (b) Model confidence; (c) In general, the predicted local-distance difference test (pLDDT) for intra-domain confidence; (d) Predicted aligned error (PAE) for determining between-domain or between-chain confidence; (e) peptide helix predicted by AlphaFold; (f) peptide helix predicted by PEP-FOLD3; Tables S1–S4: Prediction reports of the binding pocket for ligand and amino acid composition for four ligands. Tables S5–S8: PEP-FOLD clustering reports for each peptide sequence.

## Sample Availability

Not available.
