# Supplementary material for: Rational Design of Peptides Derived from Odorant-Binding Proteins for SARS-CoV-2-Related Volatile Organic Compounds Recognition
Source: Molecules. 2022 Jun 18;27(12):3917. doi: 10.3390/molecules27123917 (PMC9229983; doi:10.3390/molecules27123917)
Supplement: Supplementary file 1 [file molecules-27-03917-s001.zip › molecules-1767228-supplementary.pdf]

Article

Rational Design of Peptides Derived from Odorant-Binding Proteins for SARS-CoV-2-Related Volatile Organic Compounds Recognition

Jin Wang *, Kenji Sakai and Toshihiko Kiwa

Faculty of Interdisciplinary Science and Engineering in Health Systems, Okayama University, 3-1-1,Tsushima-Naka, Kitaku, Okayama, 700-8530, Japan; wangjin@okayama-u.ac.jp (J.W.); sakai-k@okayama-u.ac.jp (K.S.); kiwa@okayama-u.ac.jp (T.K.)

* Correspondence: wangjin@okayama-u.ac.jp; Tel.: +81-86-251-8129

Aaegobp39 sequence was obtained from https://www.uniprot.org/uniprot/Q176B6:

MYVVNLVLVLLSLEILSTSDAAMTMKQLKNSLEMMRKACAPKFNVVEASLDELKAGRFAN
EADKELKCYTMCIAQMAGTLTKKGELSLSKTTAQIEAMLPQEIKAAAKEALNACKDIQSG
FKDPCEKVYFSAKCAAEYNPDVFFFP

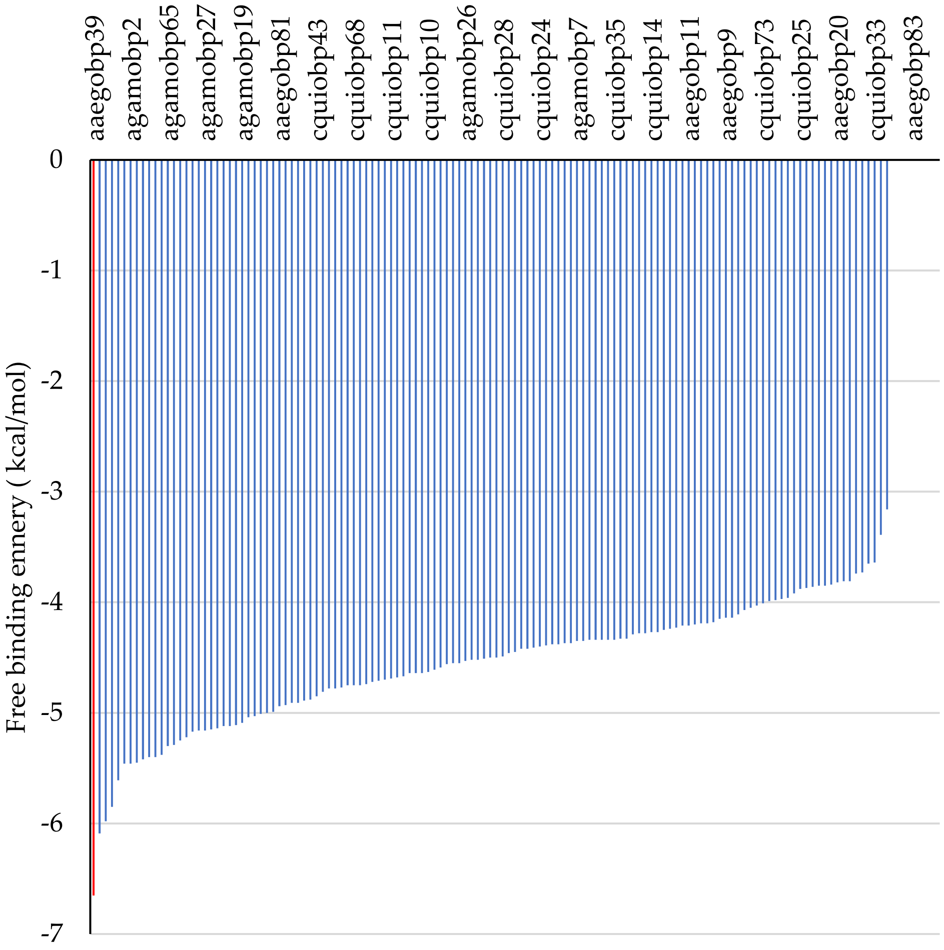

Figure S1. The free binding energy of mosquito classic and Plus-C OBPs corresponding to ligand nonanal (data was obtained from mOBP Database: http://www.bo-protscience.fr/mobpdb/)

Table S1. The prediction reports of binding pocket for ligand ethanol and the amino acids composition.

name	lig_name	volume	enclosure	surface	depth	surf/vol	hydrophobic interactions	hydrophobicity	Score
**P_0**	EOH_A_500	695.04	0.03	600.44	20.38	0.86389273	35	0.49	0.840611
**P_1**	EOH_A_500	106.69	0.15	310.6	10.04	2.91123817	9	0.25	0.278733

name	ALA	ARG	ASN	ASP	CYS	GLN	GLU	GLY	HIS	ILE
**P_0**	4	1	1	0	0	0	1	1	2	1
**P_1**	1	0	1	1	0	1	1	2	0	0

name	LEU	LYS	MET	PHE	PRO	SER	THR	TRP	TYR	VAL
**P_0**	3	0	5	4	1	4	3	1	0	5
**P_1**	0	1	0	2	0	1	0	0	0	1

Table S2. The prediction reports of binding pocket for ligand benzaldehyde and the amino acids composition.

name	lig_name	volume	enclosure	surface	depth	surf/vol	hydrophobic interactions	hydrophobicity	Score
**P_0**	HBX_A_200	552.38	0.1	540.53	20.28	0.97854738	18	0.36	0.82617
**P_1**	HBX_A_200	182.21	0.21	324.39	8.06	1.78030844	17	0.61	0.270989
**P_2**	HBX_A_200	182.08	0.09	407.51	11.39	2.23808216	16	0.37	0.365149

name	ALA	ARG	ASN	ASP	CYS	GLN	GLU	GLY	HIS	ILE
**P_0**	2	2	4	0	0	1	2	2	0	2
**P_1**	0	0	1	2	0	0	1	0	1	0
**P_2**	1	1	1	2	0	0	2	1	1	0

name	LEU	LYS	MET	PHE	PRO	SER	THR	TRP	TYR	VAL
**P_0**	5	0	0	3	0	0	0	0	4	3
**P_1**	1	1	1	1	0	0	0	1	1	0
**P_2**	2	1	0	0	0	0	1	0	2	0

Table S3. The prediction reports of binding pocket for ligand acetone and the amino acids composition.

name	lig_name	volume	enclosure	surface	depth	surf/vol	hydrophobic interactions	hydrophobicity	Score
**P_0**	Acetone	1485.44	0.13	1508.9	27.57	1.0157933	75	0.4	0.814444
**P_1**		279.23	0.15	418.17	11.09	1.49758264	21	0.51	0.489561
**P_2**		256.45	0.17	554.04	14.71	2.16042113	26	0.49	0.599924

name	ALA	ARG	ASN	ASP	CYS	GLN	GLU	GLY	HIS	ILE
**P_0**	5	1	2	0	6	3	4	6	3	5
**P_1**	1	0	0	1	0	0	1	1	1	4
**P_2**	2	0	2	0	2	0	1	1	1	0

name	LEU	LYS	MET	PHE	PRO	SER	THR	TRP	TYR	VAL
**P_0**	6	2	4	4	7	0	3	0	2	4
**P_1**	0	2	0	1	0	0	0	0	0	0
**P_2**	1	1	1	3	1	1	1	0	0	1

Table S4. The prediction reports of binding pocket for ligand nonanal and the amino acids composition.

name	lig_name	volume	enclosure	surface	depth	surf/vol	hydrophobic interactions	hydrophobicity	Score
**P_0**	Nonanal	1027.78	0.03	1033.37	23.61	1.00543891	61	0.49	0.821954
**P_1**	Nonanal	242.69	0.14	453.37	14.27	1.86810334	21	0.46	0.585585
**P_2**	Nonanal	112.06	0.26	318.06	6.11	2.83830091	15	0.47	0.183907

name	LEU	LYS	MET	PHE	PRO	SER	THR	GLY	HIS	ILE
**P_0**	6	2	5	3	1	3	3	0	0	4
**P_1**	1	2	0	3	2	0	0	1	0	0
**P_2**	1	1	0	1	1	0	0	0	0	0

name	LEU	LYS	MET	PHE	PRO	SER	THR	TRP	TYR	VAL
**P_0**	6	2	5	3	1	3	3	0	2	2
**P_1**	1	2	0	3	2	0	0	0	0	0
**P_2**	1	1	0	1	1	0	0	0	0	2

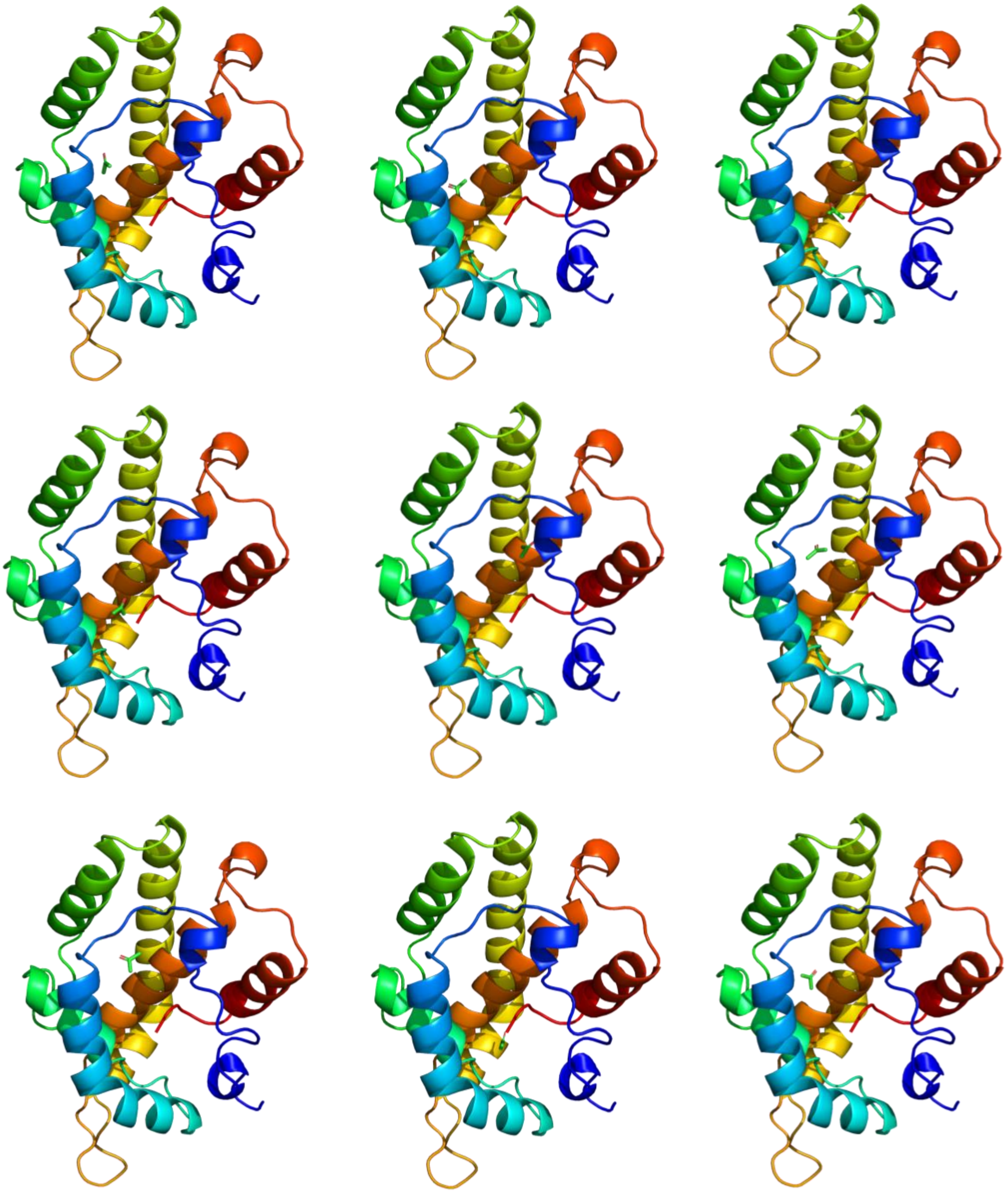

Figure S2. Nine docking modes of AgamOBP47 and ligand acetone complex.

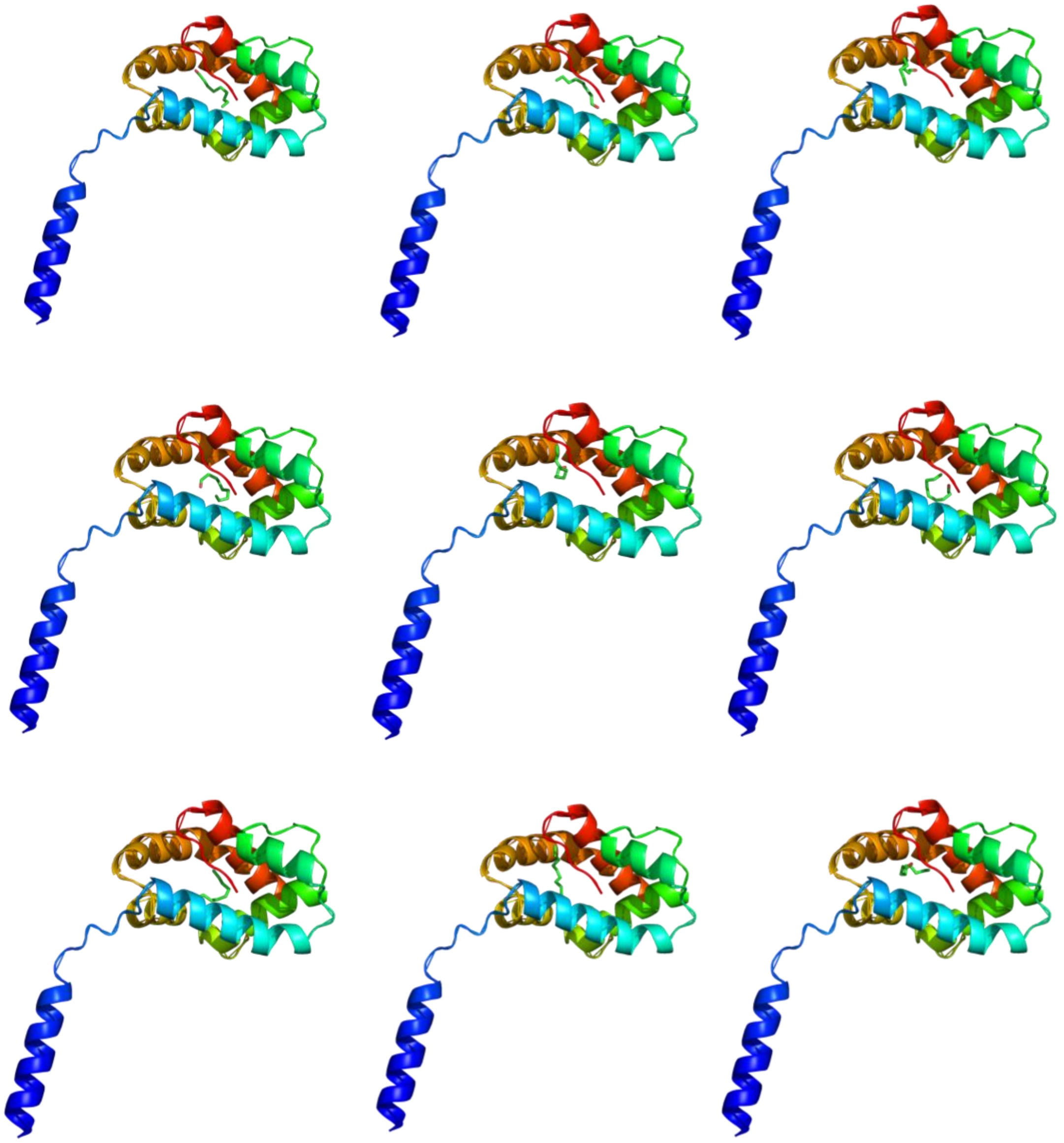

Figure S3. Nine docking modes of Aaegobp39 and ligand nonanal complex.

Table S5. PEPFOLD clustering reports (peptide sequence: TIAATLTILAALVSAANVFFF)

Model	sOPEP	avg	gdt	max	q	tm
**PEPFOLD-model1**	−44.9829	0.769	0.89	0.825	0.718	0.645
**PEPFOLD-model2**	−44.7545	0.728	0.874	0.793	0.685	0.56
**PEPFOLD-model3**	−44.4068	0.767	0.887	0.816	0.716	0.649
**PEPFOLD-model4**	−43.1849	0.739	0.832	0.798	0.682	0.642
**PEPFOLD-model5**	−43.1494	0.794	0.919	0.878	0.777	0.602

Table S6. PEPFOLD clustering reports (peptide sequence: VRGLLYVNAYAFLY)

Model	sOPEP	avg	gdt	max	q	tm
**PEPFOLD-model1**	−19.4774	0.728	0.865	0.803	0.685	0.561
**PEPFOLD-model2**	−19.464	0.649	0.766	0.643	0.613	0.573
**PEPFOLD-model3**	−19.3332	0.642	0.812	0.713	0.585	0.457
**PEPFOLD-model4**	−19.1929	0.643	0.775	0.646	0.611	0.539
**PEPFOLD-model5**	−18.9826	0.685	0.8	0.722	0.632	0.584

Table S7. PEPFOLD clustering reports (peptide sequence: TPFLVFVVYLFL)

Model	sOPEP	avg	gdt	max	q	tm
**PEPFOLD-model1**	−19.1114	0.442	0.629	0.489	0.375	0.275
**PEPFOLD-model2**	−18.3455	0.48	0.659	0.521	0.404	0.335
**PEPFOLD-model3**	−17.1037	0.487	0.675	0.56	0.42	0.295
**PEPFOLD-model4**	−15.848	0.541	0.698	0.62	0.493	0.353
**PEPFOLD-model5**	−15.5831	0.542	0.707	0.626	0.499	0.337

Table S8. The PEPFOLD clustering reports (peptide sequence: SATVFVTFW)

Model	sOPEP	avg	gdt	max	q	tm
**PEPFOLD-model1**	−6.28941	0.645	0.817	0.728	0.597	0.439
**PEPFOLD-model2**	−6.26337	0.626	0.809	0.715	0.592	0.389
**PEPFOLD-model3**	−6.24768	0.631	0.829	0.738	0.572	0.386
**PEPFOLD-model4**	−5.46783	0.633	0.822	0.733	0.587	0.392
**PEPFOLD-model5**	−5.44114	0.673	0.841	0.792	0.662	0.399

sOPEP: the coarse grained energy of PEP-FOLD; gdt: the predicted GDT_TS; q: the predicted Qmean score; tm: the predicted TMscore.

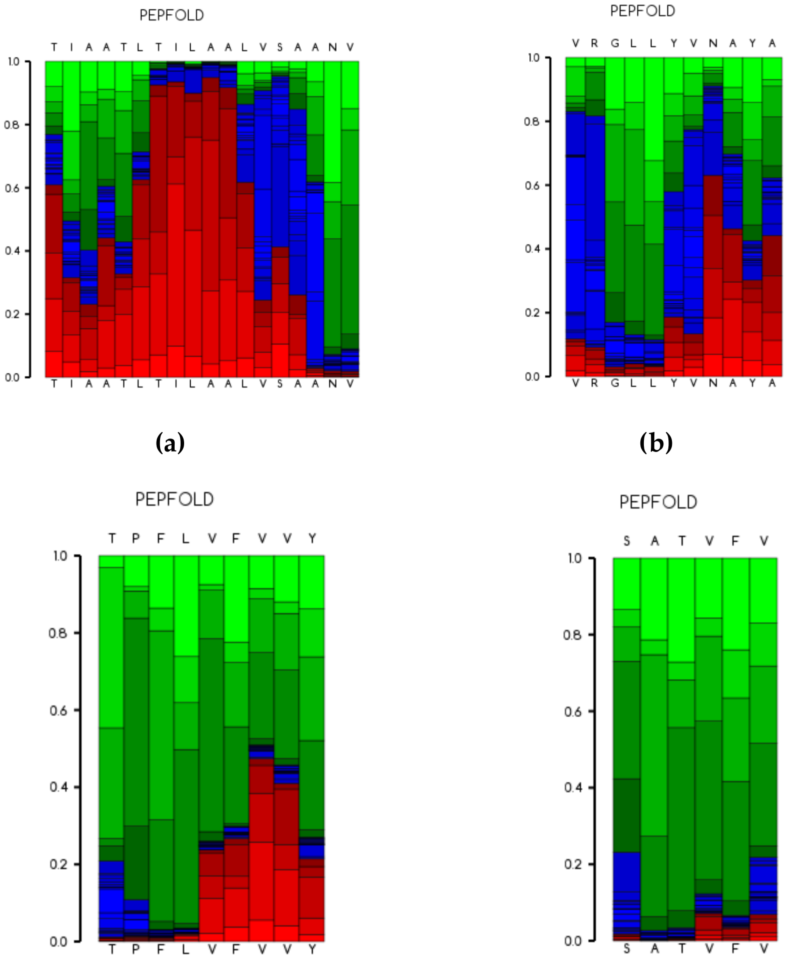

(a) (b)

(c) (d)

Figure S4. Local structure prediction profile for TIAATLTILAALVSAANVFFF(a); VRGLLYVNAYAFLY (b); TPFLVFVVYLFL (c); SATVFVTFW (d). (The profile is presented using the following color code: red: helical, green: extended, blue: coil)

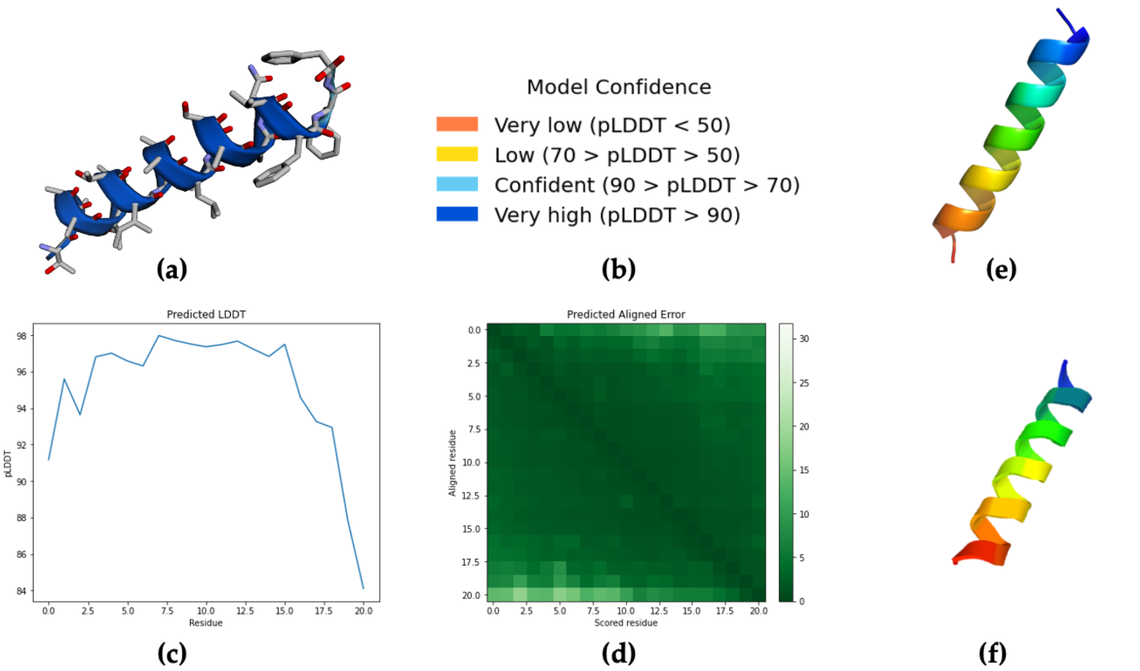

Figure S5. (a) Prediction results of nonanal binding peptide by AlphaFold Colab; (b) Model confidence; (c) In general the predicted local-distance difference test (pLDDT) for intra-domain confidence; (d) Predicted Aligned Error (PAE) for determining between domain or between chain confidence; (e) Peptide helix predicated by AlphaFold; (f) Peptide helix predicated by PEPFOLD3.
